# Modification of fatty acid selectivity of *Candida antarctica* lipase A by error-prone PCR

**DOI:** 10.1007/s10529-017-2299-0

**Published:** 2017-03-09

**Authors:** Dagmara Głód

**Affiliations:** 1 Department of Food Biotechnology, University of Warmia and Mazury in Olsztyn, Heweliusz 1 Str., 10-718 Olsztyn, Poland; 2 Department of Pathophysiology, University of Warmia and Mazury in Olsztyn, al. Warszawska 30, 10-082 Olsztyn, Poland

**Keywords:** Conjugated linoleic acid, Directed molecular evolution, Error-prone PCR, Lipase, Protein engineering, Selectivity

## Abstract

**Objective:**

To generate *Candida antarctica* lipase A (CAL-A) mutants with modified fatty acid selectivities and improved lipolytic activities using error-prone PCR (epPCR).

**Results:**

A *Candida antarctica* lipase A mutant was obtained in three rounds of epPCR. This mutant showed a 14 times higher ability to hydrolyze triacylglycerols containing conjugated linoleic acids, and was 12 and 14 times more selective towards *cis*-9, *trans*-11 and *trans*-10, *cis*-12 isomers respectively, compared to native lipase. Lipolytic activities towards fatty acid esters were markedly improved, in particular towards butyric, lauric, stearic and palmitic esters.

**Conclusion:**

Directed molecular evolution is an efficient method to generate lipases with desirable selectivity towards CLA isomers and improved lipolytic activities towards esters of fatty acids.

**Electronic supplementary material:**

The online version of this article (doi:10.1007/s10529-017-2299-0) contains supplementary material, which is available to authorized users.

## Introduction

Directed molecular evolution is used for improving or altering the activity of enzymes. It has two basic requirements: to efficiently introduce random mutations into a gene of interest and to create an effective library of variants (Copp et al. 2014).

One of the most frequently used methods of the directed evolution is error-prone PCR (epPCR). It allows engineering of improved enzymes by random changes in the nucleotide sequence using thermostable polymerases lacking proofreading ability. The polymerase error rate is controlled by addition of Mn^2+^, the number of thermocycles, and manipulation of concentrations of template and of the dNTP (Nannemann et al. 2011).

Application of random mutagenesis does not require knowledge of the enzyme structure, but needed well-chosen selection and high-throughput screening systems. Mutants with the best features are then subjected to the next rounds of mutations and selections (Chen 2001). Directed molecular evolution may however result in the disadvantageous changes in the protein structure due to the random nature of mutations, and the large libraries of mutants may result in the poor chances of obtaining enzyme with the desired properties (Chica et al. 2005).

Due to their specific properties and the large spectrum of applications in the food, pharmaceutical and cosmetic industries, lipases (E.C. 3.1.1.3) are of interest as important biocatalysts. Utility of lipases is based on their selectivity towards fatty acids, particularly towards their *cis* and *trans* isomers and conjugated *cis*/*trans* dienes of CLA. CLA exhibits unique health-supporting properties, including anti-tumor, anti-diabetic, anti-atherosclerotic and immunomodulatory (Warwel and Borgdorf 2000). Not all, however, isomers of CLA have comparable physiological effects on human health, and therefore evaluation of the biological activity of each of the produced isomers is of crucial importance.

## Materials and methods

### Culture conditions, amplification and cloning of a lipase A gene from *C. antarctica*

*Candida antarctica* ATCC 28323 was grown in YPD medium with 1% (w/v) peptone, 2% (w/v) yeast extract and 2% (w/v) glucose, pH 4.5. Submerged cultivation was carried out at 30 °C for 72 h, at 300 rpm in a 300 ml Erlenmeyer flask. Genomic DNA (gDNA) was isolated using QIAamp DNA Mini Kit (Qiagen). Primers for the lipase A gene PCR (Supplementary Table 1) were designed as recommended by pETBlue-2 (Novagen). PCR products were separated by electrophoresis on 1% (w/v) agarose gel. Cloning was performed using Perfectly Blunt Cloning Kits (Novagen). Plasmid DNA was isolated from the NovaBlue Singles Competent Cells using QIAprep Miniprep (Qiagen), and a clone expressing recombinant protein was selected from transformed *E. coli* Tuner (DE3)p*Lac*I competent cells. Plasmid DNA was isolated and the lipase A gene was sequenced using a Sanger DNA sequencing method. This plasmid DNA was used as a template in the epPCR reactions.

### Error-prone PCR (epPCR)

Random mutations of the lipase A gene were performed using GeneMorph II Random Mutagenesis Kit (Stratagene). Characteristics of introduced varied-frequency mutations are shown in Supplementary Table 2. The epPCR conditions were: initial denaturation at 95 °C for 2 min followed by 22 (low mutation frequency) or 30 rounds (medium mutation frequency) of denaturation at 94 °C for 30 s, annealing at 55 °C for 30 s, extension at 72 °C for 1 min and a final extension at 72 °C for 10 min. Modified genes were cloned into the pETBlue-2 vector, plasmid DNA was isolated from the *E. coli* NovaBlue Singles cells and transformed into the *E.coli* Tuner (DE3)p*Lac*I competent cells.

### Analysis of mutant libraries for the lipolytic activity using agar diffusion method

Mutants were grown on agar-stabilized lysogeny broth (LB) with 1% (w/v) glucose, antibiotics 50 μg carbenicillin/ml and 34 μg chloramphenicol/ml, 1 mM IPTG, and 3% (w/v) emulsion of tributyrin or triolein. Zones of hydrolyzed substrates were assessed after 48–72 h.

### High-throughput expression in *E. coli* in a microplate format

Expression in microplates was induced with 1 mM IPTG. After 3 h cultures were transferred to the test tubes containing zirconia/silica beads and homogenized using FastPrep (MP Biomedicals). Supernatants were harvested by centrifugation and used to determine the protein content (Qubit, Invitrogen), lipolytic activity, and the selectivity towards CLA isomers.

### Lipase activity and selectivity assays in a microplate format

Lipolytic activity was determined as described by Winkler and Stuckman (1979). *p*-nitrophenyl esters (*p*-NP) of fatty acids were assayed in 0.1 M phosphate buffer pH 7.5 containing 10 mM 2-propanol. The hydrolysis kinetic was determined by measurement of the released *p*-nitrophenyl at 410 nm, at 37 °C, at 10 min intervals. One unit (U) of enzyme activity was defined as the amount of enzyme required to hydrolyze 1 μmol substrate per min per mg protein. Activities are expressed in units per mg protein. Substrate selectivity was determined using *p*-NP of elaidic and vaccenic acids (*trans*), and of oleic acid (*cis*). The 1 mM *p*-NP esters were dissolved in 90 ml 50 mM phosphate buffer pH 7.5 containing 10 ml 2-propanol and 100 mg gum Arabic. The reactions were carried out as above.

### Determination of the *trans*/*cis* fatty acid selectivity

For the TAG-CLA hydrolysis reactions, 100 μl culture supernatants in 100 μl phosphate buffer pH 7, and 50 μl triacyloglycerols containing CLA (TAG-CLA; Natural ASA, Norway) were used. The reaction mixture was incubated at 30 °C for 24 h. Samples containing released fatty acids were methylated with trimethylsilyldiazomethane, (TMS). A sample of TAG-CLA was also methylated to generate a standard for evaluation of lipase A mutants for their selectivity toward *trans*/*cis* isomers of fatty acids. Chromatographic separation of the TAG-CLA sample showed the presence of the following fatty acids: *cis*-9, *trans*-11; *trans*-10, *cis*-12; *cis*-9, *cis*-11; *cis*-10, *cis*-12, *trans*-9, *trans*-11 and *trans*-10, *trans*-12 (Supplementary Fig. 1). Total composition of isomers was determined using the modified Peisker method (Peisker 1964; Zegarska et al. 1991). Fatty acid were analysed by gas chromatography using a Supelcowax 10 (30 m × 0.32 mm × 0.25 µm) column and an FID detector. Chromatogram analyses were carried out using a TotalChrom (Perkin Elmer) program.

### Characterization of selected modified lipases

Samples of selected mutants were collected after 3 and 12 h growth following induction. Cultures were pelleted by centrifugation, washed, resuspended in 0.1 M phosphate buffer pH 7.5 and homogenized. Extracellular and intracellular protein content and the lipolytic activities towards selected fatty acids were determined in supernatants obtained after (a) mutant culture pelleting, and (b) centrifugation of homogenized mutant cultures. Proteins in supernatants were separated by SDS-PAGE and gels were analyzed using Kodac Gel Logic 200. Lipolytic activities were characterized by zymography.

### Protein electrophoresis

Protein separation was performed using SDS-PAGE gels, stained with Coomassie Brilliant Blue R-250 and analyzed using Kodak Gel Logic 200.

### Zymography of lipolytic enzymes

Proteins were renatured by 2 h incubation in a Triton X-100 (0.5% w/v in 0.1 M Tris/HCl buffer, pH 7.5), and the gel was then incubated in a mixture of freshly prepared solutions A and B (1:1) for 2 h. Solution A: 50 ml of 1 M Tris/HCl pH 7.5 containing 20 mg α-naphtyl acetate in 5 ml acetone; solution B: 50 ml of 1 M Tris/HCl pH 7.5 containing 50 mg Fast Red TR salt.

## Results and discussion

### Cloning of CAL-A

Cloning and selection in the NovaBlue Singles cells yielded 37 positive colonies containing inserted gene. One of the mutants contained plasmid DNA of the 1389 base pairs gene size. Sequencing confirmed the correctness of the construct, i.e. proper sequence and the insert orientation in the pETBlue-2 vector. The amplified sequence was compared with the reference sequence of lipase A (Svendsen et al. 1998) and was performed using both the CLC Main Workbench and the BLAST search that gave identical results. Supplementary Fig. 2 shows the diagram generated by the former method. The sequence comparison revealed 30 nucleotide deviations corresponding to 10 alterations in the amino acid residues. The altered amino acids were, however, located outside of the hydrophobic tunnel, so these changes are not likely to have had an effect on the original catalytic activity of the cloned CAL-A. The observed deviations were likely associated with the genomic differences between the two strains, one reported in the Svendsen et al. (1998), and the second one used in the amplification reaction.

### Lipase A gene modification using epPCR

We have used low and medium mutation frequency of the lipase A gene based on our previous finding that the high mutation frequency resulted in the inactivation of the encoded enzyme. The obtained here epPCR products had an appropriate DNA concentration and the optimal ratios of the sample purity in each reaction.

Cloning and selection of genes modified in the first epPCR round of a low mutation frequency yielded 640 positive and 70 negative mutants what represented 90% of the ligation efficiency. Using medium mutation frequency, 400 positive and 50 negative mutants were obtained, what corresponded to the 89% of the ligation efficiency. In the second and third epPCR round of a medium mutation frequency, there were approximately 5000 and approx. 5000 positive mutants, and 150 and 132 negative mutants obtained, respectively. Such low ligation yield (approx. 50%) in the subsequent epPCR rounds could result from the accumulation of point mutations leading to the lower construct stability in *E. coli*.

### Selection and screening of mutant libraries following epPCR

**Fig. 1:**
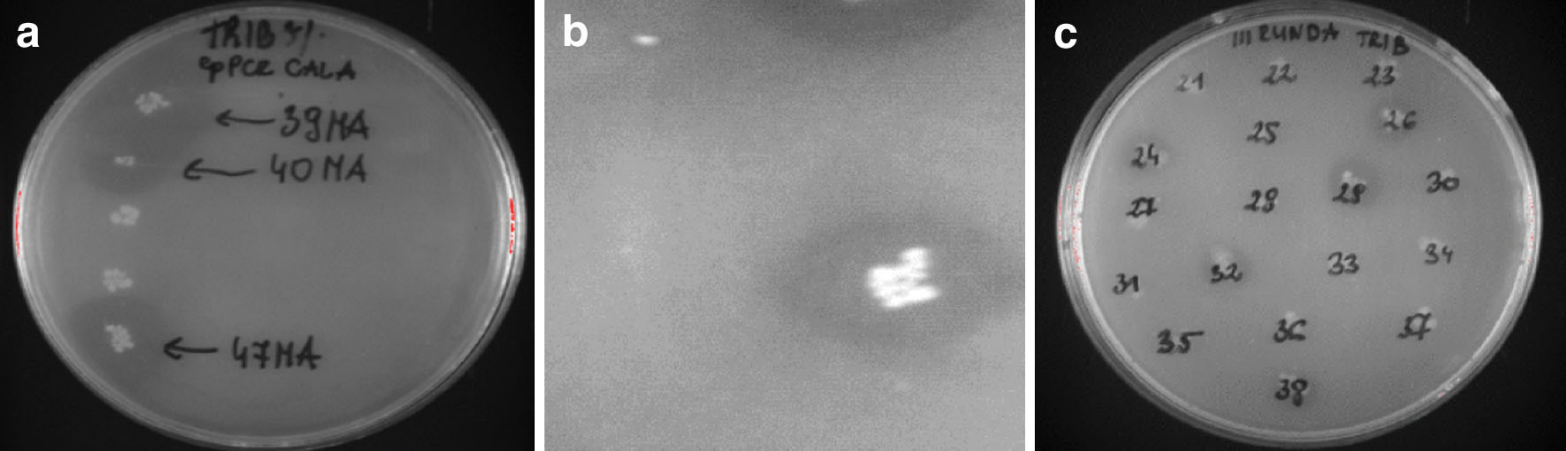
Examples of mutant cultures grown on the agar-stabilized medium, after selection using diffusion method (MA—medium mutation frequency): **a** the second round of epPCR, **b** wild-type lipase and **c** the third round of epPCR. Colonies with halo-forming activity on tributyrin/triolein agar plates were isolated

Following isolation of plasmid DNA and transformation into the Tuner (DE3)p*Lac*I expressing cells the obtained mutants were plated on agar medium supplemented with antibiotics and 3% (w/v) of the emulsion of a short-chain tributyrin or a long-chain triolein to select these mutants, which were able to hydrolyze at least one substrate. Most of mutants hydrolyzed tributyrin, and a small proportion of mutants hydrolyzed either substrate or only triolein. The native lipase A Fig. 1 and mutant MA39 hydrolyzed only tributyrin. Representative agar diffusion images are shown in Fig. 1. Colonies of mutants hydrolyzing at least one substrate were used in the next step, i.e. culturing in the high-yield microplate.

**Fig. 2:**
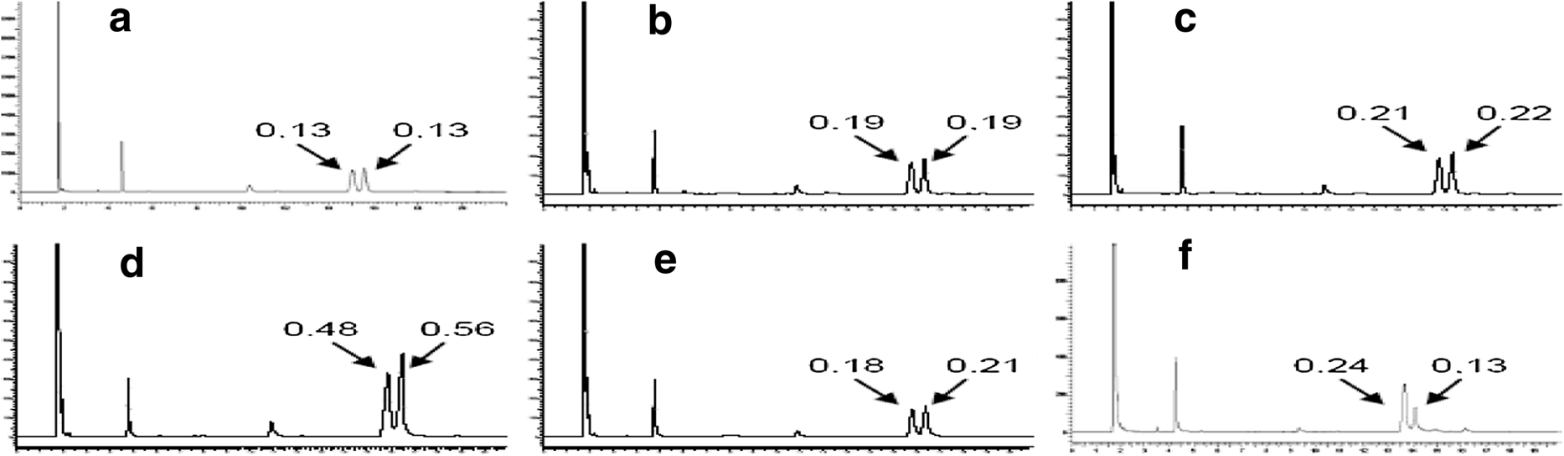
Chromatograms of selected samples following TAG-CLA substrate hydrolysis and methylation of the released fatty acids (LA—low mutation frequency; MA—medium mutation frequency): **a** MA 39, **b** LA 133, **c** LA 140 **d** LA 163, **e** LA 168 and **f** Novozym 735. *Arrows* indicate isomers *cis*-9, *trans*-11 and *cis*-10, *trans*-12 with a selectivity constant α values

The MA39 clone obtained with a medium mutation frequency in the first epPCR round showed selectivity for *cis*-9, *trans*-11 (a = 0.13) and *trans*-10, *cis*-12 (a = 0.13). The degree of TAG-CLA hydrolysis by the MA39 clone was 8.8%. For comparison, the TAG-CLA hydrolysis was carried out using native lipase and a commercial enzyme preparation Novozym 735. Novozym 735 was characterized by the selectivity towards the *cis*-9, *trans*-11; *trans*-10, *cis*-12; *cis*-10, *cis*-12 and *trans*-9, *trans*-11 & *trans*-10, *trans*-12, and the selectivity constant α for these isomers was 0.24; 0.13; 0.21 and 0.01, respectively. The degree of TAG-CLA hydrolysis by the Novozym 735 was about 13%. The selectivity ratio of the *C. antarctica* A native lipase towards the *cis*-9, *trans*-11 and *trans*-10, *cis*-12 was a = 0.02, and the degree of TAG-CLA hydrolysis was 1.2%. Table 1 shows the selection of mutants having a most favorable capacity for TAG-CLA hydrolysis, and chromatograms showing resolved fatty acids following substrate hydrolysis and methylation are shown in Fig. 2.

**Table 1:** Comparison of the selectivity of designated lipases towards TAG-CLA after the first epPCR round

Clones	Selectivity constant α towards CLA isomers					
	*Cis*-9, *trans*-11	*Trans*-10, *cis*-12	*Cis*-9, *cis*-11	*Cis*-10, *cis*-12	*Trans*-9, *trans*-11 & *trans*-10, *trans*-12	Degree of TAG-CLA hydrolysis β (%)
MA39-I	0.13	0.13	–	–	–	8.8
LA133	0.19	0.19	–	–	–	12.4
LA140	0.21	0.22	–	–	–	14.4
LA163	0.48	0.56	0.57	–	0.01	35.0
LA168	0.18	0.21	–	–	–	12.6
Novozym 735	0.24	0.13	–	0.21	0.01	13.2

*MA* medium mutation frequency, *LA* low mutation frequency

**Fig. 3:**
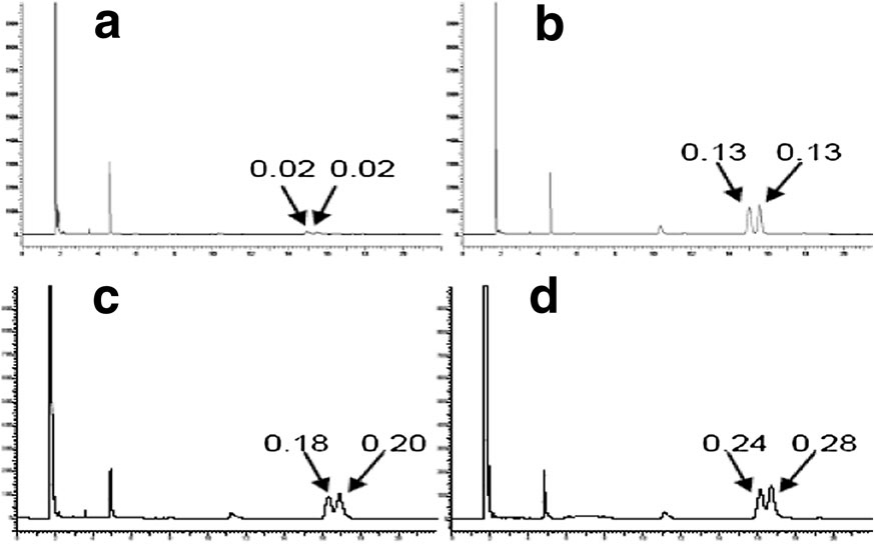
Chromatogram of the TAG-CLA hydrolysis products of the selected MA39 mutant, after subsequent epPCR rounds: **a** wild-type lipase, **b** first round, **c** second round and **d** third round. *Arrows* indicate isomers *cis*-9, *trans*-11 and *cis*-10, *trans*-12 with a selectivity constant α values

Subsequent second and third epPCR rounds of mutant MA39 allowed selection of a variant with the 14-fold increased TAG-CLA hydrolysis activity relative to the native lipase, from 1.2 to 17.1%. This mutant has also shown 12- and 14-fold increased selectivity for *cis*-9, *trans*-11 and *trans*-10, *cis*-12, respectively, as compared to the native lipase (Table 2). The results of the chromatographic analysis of the mutant MA39 activity following subsequent epPCR rounds are shown in Fig. 3.

**Table 2:** Differences between MA39 mutant and the native lipase in the selectivity constant α towards CLA *trans*/*cis* isomers, and in the degree of TAG-CLA hydrolysis β

Clones	Selectivity constant α towards CLA isomers					
	*Cis*-9, *trans*-11	*Trans*-10, *cis*-12	*Cis*-9, *cis*-11	*Cis*-10, *cis*-12	*Trans*-9, *trans*-11 & *trans*-10, *trans*-12	Degree of TAG-CLA hydrolysis β (%)
Wild-type lipase	0.02	0.02	–	–	–	1.2
MA39-I	0.13	0.13	–	–	–	8.8
MA39-II	0.18	0.20	–	0.03	–	12.4
MA39-III	0.24	0.28	–	0.03	0.01	17.1

The activity of CAL-A is higher towards substrates containing short- and medium-chain fatty acids compared to long-chain fatty acids (Adamczak 2003). Interestingly, the unique construction of the acyl tunnel of lipase A determines a perfect fit for long-chain fatty acids and also affects the selectivity towards *trans*-fatty acids (Brundiek et al. 2012).

**Fig. 4:**
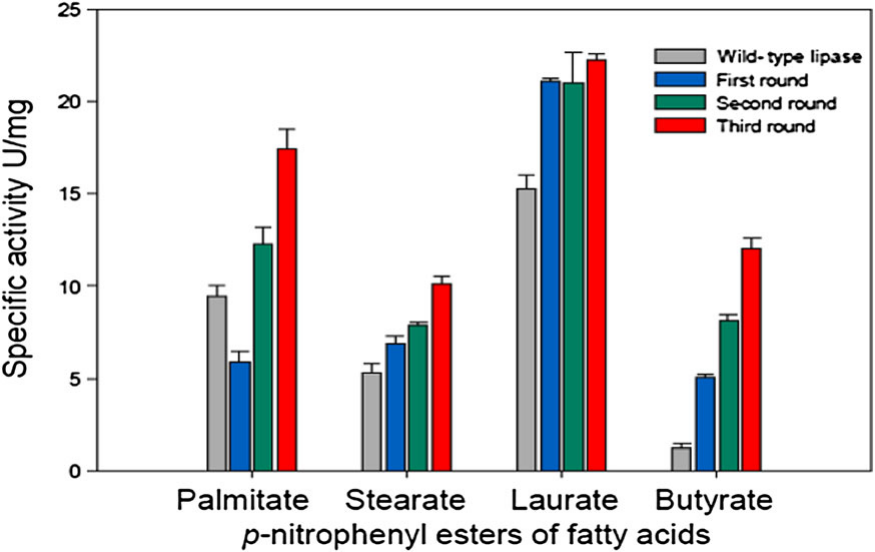
Intracellular lipase activity of the MA39 mutant towards fatty acid esters after subsequent epPCR rounds compared to the native lipase activity in the Tuner (DE3)p*Lac*I cells (submerged cultivation with shaking, 37 °C, 3 h from induction); the data shown represent the averaged values from three experiments ± SD

Both native lipase A and the MA39 mutein had the most favorable intracellular lipolytic activity towards medium-chain lauric acid ester, but the greatest difference in the lipolytic activity was observed towards short-chain butyric acid, with the MA39 mutein showing much higher activity over the native lipase A. After three rounds of epPCR, the lipolytic activity increased towards butyric acid, from 1.2 to 12.05 U/mg, lauric acid: from 15.3 to 22.2 U/mg, stearic acid: from 6.8 to 10.1 U/mg and palmitic acid: from 5.8 to 17.4 U/mg. The lipolytic activity of the MA39 mutant towards esters of selected fatty acids following subsequent epPCR rounds is presented in Fig. 4.

We have determined the lipolytic activity towards the *p*-NP esters of *cis*-oleic and *trans*-elaidic acids of the entire library of mutants obtained in the third epPCR round and of the native lipase A. For comparison, we have also determined the lipolytic activities of commercial formulations of lipase A (Novozym 735, Novozymes) and of lipase B (Novozym 435, Novozymes) towards these substrates. None of the mutants including MA39 showed favorable lipolytic activity towards esters of *cis*-oleic and *trans*-elaidic fatty acids: the activities were at the level of hundredths or tenths U/mg. Therefore, to assess the substrate preferences we have determined the ratio of the lipolytic activity towards the *trans*-elaidic acid ester the lipolytic activity towards the *cis*-oleic acid ester. The ratio A_*trans*_/A_*cis*_ above 1 indicates the preference for hydrolysis of *trans*-elaidic acid ester (Table 3).

**Table 3:** Comparison of the lipolytic activity (U/mg) towards esters of *cis*-oleic and *trans*-elaidic fatty acids between the selected MA39 mutant and the native lipase in the Tuner (DE3)*pLac*I cells (submerged cultivation with shaking, 37 °C; E—extracellular activity, I—intracellular activity, and the commercial lipase preparations; the data shown represent the averaged values from three experiments ± standard deviation

Clones/enzymatic preparations	*P*-nitrophenyl esters		Selectivity constant		Selectivity constant A_*trans*_/A_*cis*_	
	Oleic acid (*cis*)		Elaidic acid (*trans*)			
	E	I	E	I	E	I
Wild-type lipase	0.012 ± 0.001	0.015 ± 0.001	0.014 ± 0.001	0.021 ± 0.003	1.17	1.40
MA39-I	0.051 ± 0.002	0.030 ± 0.001	0.137 ± 0.003	0.077 ± 0.003	2.70	2.56
Novozym 435 (CAL-B)	2.63 ± 0.10		3.76 ± 0.25		1.42	
Novozym 735 (CAL-A)	5.00 ± 0.30		15.00 ± 0.60		3.00	

### Protein analysis

**Fig. 5:**
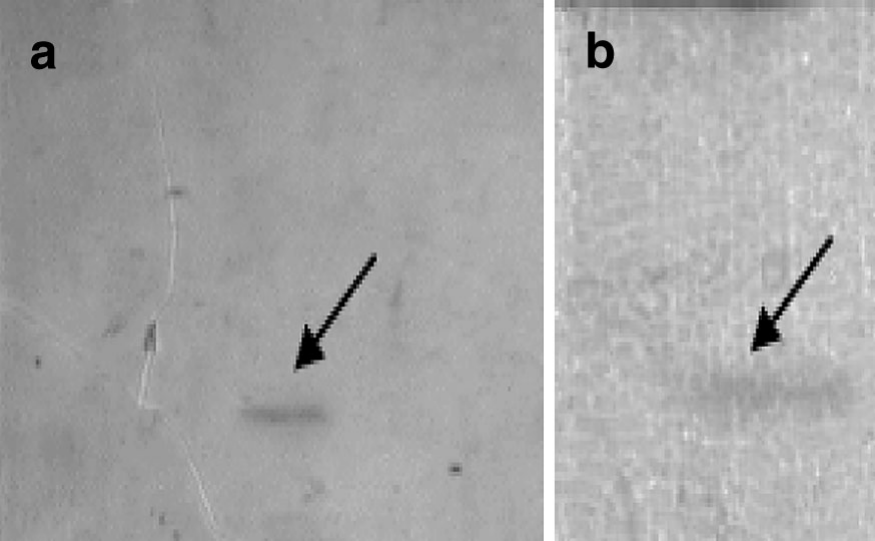
Zymogram demonstrating intracellular lipolytic activity of the MA39 mutant and of the native lipase in the Tuner (DE3)p*Lac*I cells (submerged cultivation with shaking, 37 °C, 3 h from induction); **a** MA 39; **b** wild-type lipase. *Arrows* point to the *red band* indicating lipase activity

The molecular weight of the native lipase A is 45 kDa (Patkar et al. 1993). We have shown that the lipase A and mutein produced in the Tuner (DE3)p*Lac*I cells are of a molecular weight about 43 kDa and are secreted as confirmed by SDS-PAGE and zymography. Active lipases have the ability to hydrolyze β-naphthyl acetate to β-naphthol and acetic acid. Released β-naphthol in the presence of diazonium salt forms an insoluble product showing as red bands, the intensity of which depends on the activity of the enzyme. Figure 5 presents an example of zymogram demonstrating lipolytic activity.

**In conclusion**, Molecular evolution has yielded libraries of lipase A mutants with several-fold improved enzymic properties. These included TAG-CLA hydrolysis, lipolytic activity towards esters of short-, medium- and long-chain fatty acids and altered selectivity for CLA isomers: *cis*-9, *trans*-11 and *trans*-10, *cis*-12. Since these isomers are the main components of the CLA beneficial to human health and present in foods (Sehat et al. 1998), such approach may prove practical in the development of improved food products. We have also shown that the frequency of mutations determined the enzyme properties, with medium mutation frequency being most effective in improving the activity and selectivity of the lipase mutein.

## Electronic supplementary material

Below is the link to the electronic supplementary material.

Supplementary material 1 (DOCX 897 kb)
