# Supplementary material for: Modification of fatty acid selectivity of Candida antarctica lipase A by error-prone PCR
Source: Biotechnol Lett. 2017 Mar 9;39(5):767–73. doi: 10.1007/s10529-017-2299-0 (PMC5409807; doi:10.1007/s10529-017-2299-0)
Supplement: Supplementary file 1 — Supplementary material 1 (DOCX 897 kb) [file 10529_2017_2299_MOESM1_ESM.docx]

Supplementary Table 1 Nucleotide sequences of primers used

**Supplementary Table 2** Characteristics of the applied mutations

**Supplementary Figure 1** Chromatogram of the CLA isomers: **a** *cis*-9, *trans*-11, **b** *trans*-10, *cis*-12, **c** *cis*-9, *cis*-11, **d** *cis*-10, *cis*-12, **e** *trans*-9, *trans*-11 & *trans*-10, *trans*-12

**
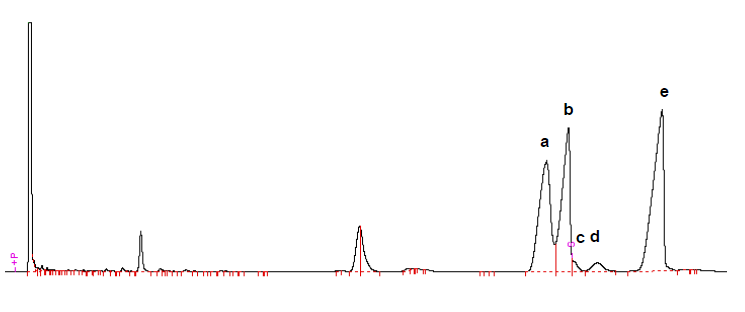
**

**Supplementary Figure 2** Sequence alignment of the amplified lipase A from the *C. antarctica* strain ATCC 28323 with the reference CAL-A

**
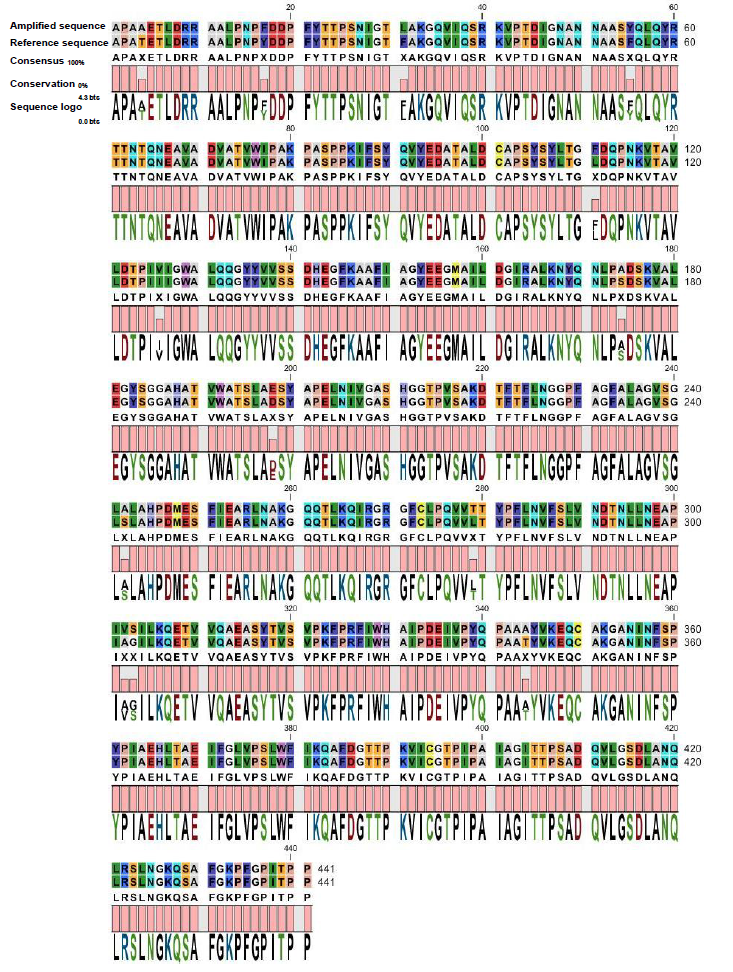
**
